# Methodological Considerations in the Kinematic and Kinetic Analysis of Human Movement among Healthy Adolescents: A Scoping Review of Nonlinear Measures in Data Processing

**DOI:** 10.3390/s23010304

**Published:** 2022-12-28

**Authors:** Sandra Silva, Fernando Ribeiro, Vânia Figueira, Francisco Pinho

**Affiliations:** 1 Escola Superior de Saúde do Vale do Ave, Cooperativa de Ensino Superior Politécnico e Universitário, Rua José António Vidal, 81, 4760-409 Vila Nova de Famalicão, Portugal; 2 School of Health Sciences, University of Aveiro, 3810-193 Aveiro, Portugal; 3 Department of Medical Sciences, University of Aveiro, 3810-193 Aveiro, Portugal; 4 Institute of Biomedicine—iBiMED, University of Aveiro, 3810-193 Aveiro, Portugal; 5 Research Centre in Physical Activity, Health and Leisure, Faculty of Sport, University of Porto, Rua Dr. Plácido da Costa, 91, 4200-450 Porto, Portugal

**Keywords:** nonlinear variables, adolescents, out-of-laboratory, variability, biomechanics

## Abstract

Nonlinear measures have increasingly revealed the quality of human movement and its behaviour over time. Further analyses of human movement in real contexts are crucial for understanding its complex dynamics. The main objective was to identify and summarize the nonlinear measures used in data processing during out-of-laboratory assessments of human movement among healthy adolescents. Summarizing the methodological considerations was the secondary objective. The inclusion criteria were as follows: According to the Population, Concept, and Context (PCC) framework, healthy teenagers between 10 and 19 years old that reported kinetic and/or kinematic nonlinear data-processing measurements related to human movement in non-laboratory settings were included. PRISMA-ScR was used to conduct this review. PubMed, Science Direct, the Web of Science, and Google Scholar were searched. Studies published between the inception of the database and March 2022 were included. In total, 10 of the 2572 articles met the criteria. The nonlinear measures identified included *entropy* (n = 8), *fractal analysis* (n = 3), *recurrence quantification* (n = 2), and the *Lyapunov exponent* (n = 2). In addition to *walking* (n = 4) and *swimming* (n = 2), each of the remaining studies focused on different motor tasks. Entropy measures are preferred when studying the complexity of human movement, especially multiscale entropy, with authors also carefully combining different measures, namely entropy and fractal analysis.

## 1. Introduction

Human movement can be described as harmonious musculoskeletal synergies conditioned by a continuous interaction of multiple neural networks, making adequate and efficient motor actions possible [1,2]. Complex motor performances are the consequences of variability and flexibility, which are needed to adapt each individual to chaotic, always-changing environments [3]. Motor variability is one of the most common characteristics of human movement, and it is related to typical variations in kinetic and kinematic patterns during the repetition of a task [4,5,6]. It is via variability that healthy biological systems are able to adjust properly in an unpredictable and constantly changing environment [7,8].

The scientific literature reports linear and nonlinear approaches for processing the kinetic and kinematic data of human movement [9]. Although they complement each other, it is known that nonlinear models reveal more of the quality of movement and the behaviour of movement over time [3]. On the other hand, linear models, although useful, seem to be insufficient for describing the characteristics of movement in human systems endowed with complexity, non-linearity, and variability [10].

Stability and complexity are two parameters that allow the assessment of motor variabilities [10]. Both can be analysed using different nonlinear measures, such as the Largest Lyapunov Exponent (LLyE), the Maximum Floquet multiplier, and fractal measures, for the stability analysis and entropy measures for the evaluation of complexity [10,11]. In 2009, Harbourne and Stergiou [9] suggested LLyE as the most frequently used nonlinear variable for measuring dynamic stability and entropy measures for evaluating the degree of an irregularity related to the complexity of movement. On the other hand, Costa et al., in 2013 [12], systematically reviewed, for the first time, studies that used nonlinear measures in the processing of kinetic and/or kinematic data among participants up to the age of 14, and they identified approximate entropy as the nonlinear measure that is most frequently used. It is important to point out that they reinforced the difficulty in defining the most appropriate measure due to the study’s variability (e.g., the tasks studied). Another important factor that can be considered is the environment in which each subject is assessed. Usually, studies are performed in laboratory facilities and in controlled environments to be more precise in measurements, but currently, with the advent of wearable and ambulatory health monitoring devices, it is possible to conduct studies outside the laboratory with a high degree of precision [13]. It is in the real-life context that problems arise, and probably, it is in that environment that solutions must be found in order to solve them in an integrated and adjusted manner relative to each individual in each motor development stage.

Movement complexity and variability may also differ depending on the stages of human development. For example, the motor learning tasks that are the foundation for all functional motor tasks are acquired at an early age [14,15]. Despite this fact, adolescence (the growth spurt phase)—characterized by a rapid increase in body mass and changes in physiological and psychological aspects related to puberty—is also associated with the exploration, experimentation, and initiation of behaviours that are determinants of health throughout life [16]. These aspects suggest the use of a combination of several measures for a global assessment of motor variability, in which the focus is the exploratory nature of the movement, with an emphasis on the quality of motor performance [3].

Therefore, the main objective of this scoping review of the literature was to summarize the nonlinear measures used in the analysis of kinetic and kinematic data of human movement in healthy adolescents who were assessed in real-life environments. After the nonlinear measures were identified, the objective was to summarize the methodological considerations, namely the tasks under study, measurement instruments, and outcomes considered.

**Review questions**

The main review question was “What are the nonlinear measures used in processing kinematic and kinetic data in the assessment of human movement among healthy adolescents?”

The review sub-questions are listed as follows:

i. What instruments are used to collect kinematic and kinetic data in the identified studies?
ii. What kinematic and kinetic variables are considered in the identified studies?
iii. What tasks are covered in the identified studies?

## 2. Materials and Methods

This scoping review was conducted in accordance with the Preferred Reporting Items for Systematic Reviewers and Meta-Analysis extension for Scoping Reviews (PRISMA-ScR) framework [17]. The protocols, namely the review questions and methodology, are specified on Open Science Framework registries, https://osf.io/bpehv (accessed on 16 March 2022). The following Supporting Information can be downloaded at https://www.mdpi.com/article/10.3390/s23010304/s1.

### 2.1. Eligibility Criteria

The eligibility criteria were established a priori using the acronym PCC (Population, Concept, and Context) in accordance with the Joanna Briggs Institute methodology [18] (Table 1).

Studies were also eligible if they met the following criteria:

- Experimental and epidemiological study designs;
- Studies published in English, Portuguese, French and Spanish.

Studies were excluded if they had any of the following characteristics:

- Systematic, narrative, or scoping reviews to avoid the duplication of data;
- Qualitative method designs.

### 2.2. Information Source

The relevant studies were identified by searching the databases—PubMed, the Web of Science, and Science Direct—from their inception until March 2022. Google Scholar was contemplated as unpublished and grey literature. The reference lists of original research articles and reviews on the topic were manually verified to identify other eligible studies. The search strategy for PubMed was as follows: ((nonlinear measures) OR (nonlinear dynamics) OR (entropy) OR (motor variability)) AND ((adolescents) OR (children)) AND ((kinematic) OR (kinetic)). Two reviewers independently carried out the search.

### 2.3. Selection of Evidence Sources

The selection of evidence sources considered the PCC acronym, purpose, and research questions. Data were extracted by two independent reviewers, and any disagreements between them were resolved via discussions or with a third reviewer.

A pilot test was carried out where all reviewers analysed the same 25 publications (the first 25 titles/abstracts of the PubMed database) [19]. Based on eligibility criteria defined a priori, an analysis of the titles/abstracts was carried out independently by the two reviewers. The researchers started the screening process only when there was a consensus of at least 75% [19].

After the search, all identified records were imported to the Mendeley software (Elsevier), and duplicates were removed. The titles and abstracts were screened by the same two reviewers that categorized the studies as “include” or “exclude”. This stage allowed identifying articles for full-text screening.

### 2.4. Data Extraction

Data were extracted regarding the authors, year of publication, study design, characteristics of the participants (n, age, % female, and body mass), study setting, the tasks under study, assessment instruments to capture the human body motion, kinetic and kinematic variables, and nonlinear measures. The corresponding author of one of the included articles was contacted by e-mail to request additional data. Two authors independently extracted the abovementioned data by using a draft charting table adapted from the original JBI template. Any disagreements were resolved with a third author.

### 2.5. Data Presentation

A narrative report was produced to summarize the extracted data around the following outcomes: nonlinear measures, instruments, kinematic and kinetic variables, and tasks and contexts. These results were described in relation to the research question and in the context of the overall study purpose. A tabular form complemented this synthesis of the main findings.

## 3. Results

**Figure 1:**
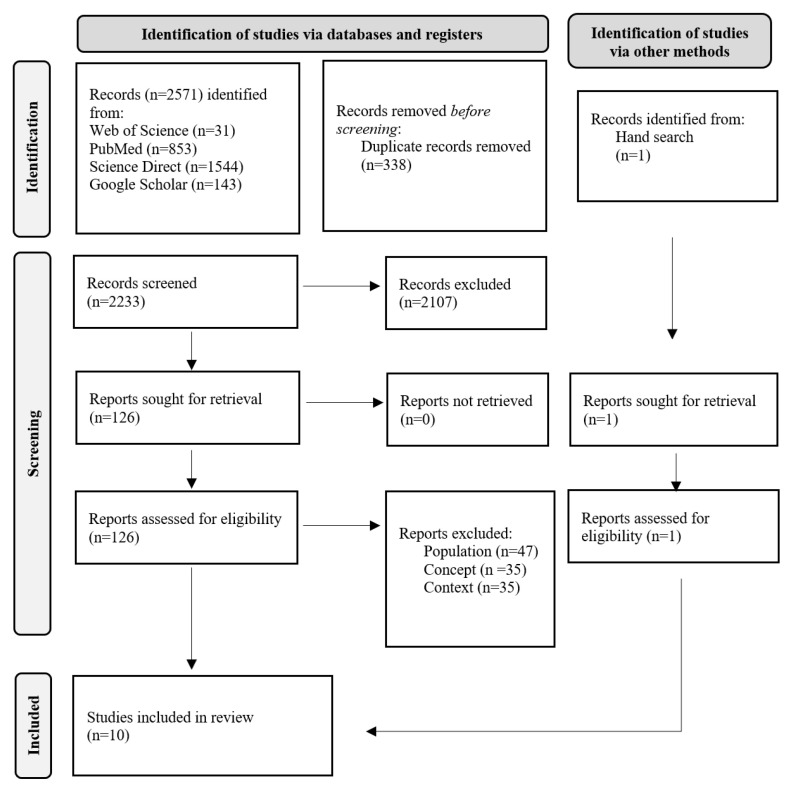
Flow diagram for the scoping review process adapted from the PRISMA-ScR statement [20].

A total of 2572 articles were identified—2571 records via a database search, and one additional article was identified via a hand search of the reference lists. After removing 338 duplicates, 2233 records remained. The screening process of the titles and abstracts led to the removal of 2107 articles, leaving 127 for full-text analyses. Of these, 117 were excluded after full-text analyses since they did not fulfil the inclusion criteria, namely the population (n = 47), concept (n = 35), and context (n = 35). Hence, 10 articles were included in this review. The study selection process is provided in the flowchart (Figure 1).

In total, 10 studies enrolled a total of 261 adolescents. The characteristics of the participants are listed in Table 2. The mean sample size was 26.1 participants, ranging from 10 to 42. In total, eight of the ten studies enrolled participants of both sexes: one study involved only male participants [21], and one study enrolled only female participants [22] (Table 2).

### 3.1. Tasks and Context

Regarding the assessed tasks (Table 2), four studies analysed walking [23,24,25,26]; of those, two studies assessed normal walking (NW) and tandem walking (TW) [25,26]. The remaining studies focused on the analysis of stair descent walking [22], a quiet upright stance [27], trunk and upper limb movement [21], swimming [28,29], and long swinging on a high bar [30]. Three studies that assessed walking [23,24,25,26] or stair descent walking [22] used a self-selected speed. The tasks were carried out in real contexts, namely schools [22,24,25,26,27], training centres [21,23,30], and aquatic environments [28,29].

**Table 2:** Characteristics of the participants, study design, study setting, and tasks.

Author, Year	Participants	Study Design	Study Setting	Tasks
Hausdorff et al., 1999 [23]	11–14 years: n = 12 50% female (F) Weight 44.4 ± 2.7 kg	Observational, analytical study	400-m Running track	Walking at their self-determined normal pace for 8 min around a running track.
Pau et al., 2012 [27]	10–11 years: n = 42 (50% obese and 50% non-obese) Non-obese: 10.5 ± 0.40 years; height 142.9 ± 7.7 cm; weight 35.5 ± 5.8 kg Obese: 10.7 ± 0.30 years; height 145.3 ± 6.7 cm; weight 48.3 ± 6.3 kg	Observational, transversal, analytical study	School	Quiet upright stance with and without its backpack in terms of a conventional COP-based measure.
Rathleff et al., 2013 [22]	16.6–17.2 years: n = 29 100% f height: 168.4 (166.4–170.5) cm weight: 59.1 (55.6–62.6) kg	Cross-sectional population-based study	School	Stair descent walking at self-selected speeds: The stairway consisted of two sets of 12 steps separated by a short landing. Subjects took approximately four steps on level ground before starting the stair descent.
Barbosa et al., 2015 [28]	F: n = 12–12.43 ± 0.78 years Boys: n = 13–12.64 ± 0.81 years	Longitudinal study	Swimming pool	Swimming a maximal 25 m front crawl trial with a push-off start.
Bisi and Stagni, 2016 [24]	10 groups of different ages (n = 10, each group), of which: 10 years: height 145.0 ± 8.0 cm; weight 40.0 ± 5 kg 15 years: height 164.0 ± 6.0 cm; weight 61.0 ± 11.0 kg	Observational, transversal, analytical study	School	Walking at a self-selected speed in a corridor longer than 12 m.
Vicinanza et al., 2018 [30]	4 groups of different ages/expertise: (n = 10 gymnasts, 100% male), of which: Junior elite (n = 5, release group): 14 ± 2.5 years; height: 155.0 ± 10.0 cm; weight: 44 ± 9 kg Junior national (n = 5, non-release group): 12 ± 3.0 years; height: 150.0 ± 10.0 cm; weight: 42.0 ± 10.0 kg	Observational, transversal, analytical study	Training Centre	Performing a series of four long swings while looped to the high bar.
Bisi and Stagni, 2018 [25]	7 groups of different ages (n = 15, each group), of which: 10 years: 7 F, 10.0 ±0 years; height 143.0 ± 8.0 cm; weight 38.0 ± 6.0 kg 15 years: 7 F, 15.0 ± 0.0 years; height 170.0 ± 7.0 cm; weight 62.0 ± 11.0 kg	Observational, transversal, analytical study	School	Walking at their self-selected speed in NW and TW back and forth along a 10 m long tapeline on the floor.
Hamacher et al., 2018 [21]	14.0 ± 1.00 years old (13–16): n = 14 (100% youth sprint kayak boy athletes) Height 178 ± 10.0 cm (158–193)	Cross-sectional study	Local Olympic Centre	Performing three minutes of paddling at each of the following stages with increased stroke rates: 62–64/min^−1^ (warm-up); 66–68 min^−1^, 72–74 min^−1^, 6–78 min^−1^, 80–82 min^−1^, and 86–90 min^−1^.
Bartolomeu et al., 2018 [29]	14.20 ± 1.71 years old (25 F): n = 49	Observational, transversal, analytical study	Swimming pool	Performing 25 m all-out sprints at front-crawl, backstroke, breaststroke, and butterfly (counterbalanced randomly assigned crossover design), each one at a full stroke (FS); only the arms’ stroke and only leg kicking in a total of 12 bouts at 6 per day.
Bisi et al., 2019 [26]	7 groups of different ages (n = 15, each group), of which: 10 years: 7 F, 10.0 ± 0 years; height 143.0 ±8.0 cm; weight 38.0 ± 6.0 kg) 15 years: 7 F, 15.0 ±0.0 years; height 170.0 ± 7.0 cm; weight 62.0 ± 11.0 kg)	Observational, transversal, analytical study	School	Walking at their self-selected speed in NW and TW back and forth along a 10 m long tapeline on the floor.

F: Female; NW: normal walking; TW: tandem walking.

### 3.2. Nonlinear Measures

The 10 studies included in this review used several nonlinear measures to analyse human movement (Table 3). Entropy measures were used in eight studies [22,24,25,26,27,28,29,30], namely sample entropy (SEn) [22,29], multiscale entropy (MSE) (calculated by assessing the SEn) [24,25,26], approximate entropy (ApE) [28], the complexity index (derived from MSE) [27], and Simpson entropy [30].

The recurrence quantification measures were used in two studies [26,30], namely the recurrence rate, determinism, and averaged diagonal line length.

To assess the local dynamic stability, two studies used the Lyapunov exponent; one used the short Lyapunov exponent (sLe) [26], and the other used the largest Lyapunov exponent (LLyE) [21], with both using Rosenstein’s algorithm.

Three studies applied fractal analyses. More specifically, the fractal dimension was assessed in one study [29] to complement the analysis with the SEn; the correlation dimension was also used in one paper [30], and a detrended fluctuation analysis (DFA) was used in one study [23] as the temporal structure measurements.

### 3.3. Instruments

Several instruments were used to assess the kinematic and kinetic variables (Table 3). Of the ten studies, one study only collected kinetic data [27], one study collected both kinetic and kinematic data [22], and the remaining eight studies only collected kinematic data [21,23,24,25,26,28,29,30].

Forces plates [27] and portable handled dynamometers [22] were used to assess the kinetic data. The muscular activity was assessed using electromyography [22]. When considering the kinematic data, it was noted that the measurement tools varied according to the context in which the adolescents were evaluated. In a school context, the studies used the electronic uniaxial goniometer [22] and two [24,25] or three axial wireless inertial sensors [26]. In the studies carried out in training centres, whether in athletics or kayak, the instruments included force-sensitive switches placed on the foot [23], six axial wireless inertial sensors [21], and a 3D motion capture system [30]. The two studies collecting data in a swimming pool used a speedometer [28,29].

### 3.4. Kinetic and Kinematic Variables

In this regard, the use of different instruments to collect human movement data led to the acquisition of different variables (Table 3). Concerning the kinetic variables, one study quantified the centre-of-pressure (COP) time series during quiet standing and provided normalized COP mean velocities in both the anteroposterior (AP) and mediolateral (ML) directions [27]. The maximal quadriceps torque and the magnitude of the muscle activity using the root mean square analysis were the main outcomes assessed in one study [22].

Of the studies assessing the kinematics of human movement, six studies described spatiotemporal parameters [21,22,23,24,25,28,29], three studies assessed both spatiotemporal and temporal parameters [22,23,28], and one study assessed only temporal parameters [26]. The joint kinematics were assessed in three studies by considering the range of motion of the knee [22,30], shoulder and elbow [21,30], hands [21], hip, ankle, and foot [30], and trunk [21].

**Table 3:** Assessment instruments, kinematic and kinetic variables, and nonlinear measures.

Author, Year	Assessment Instrument	Kinematic and/or Kinetic Variables	Nonlinear Measures
Hausdorff et al., 1999 [23]	Two force-sensitive switches (placed inside the right shoe): 1 underneath the heel and 1 underneath the ball of the foot.	Spatiotemporal parameters: Walking velocity (m/s) Temporal parameters: Stride time (s)	**Temporal structure measures:** Detrended fluctuation analysis (DFA)
Pau et al., 2012 [27]	Force plate Footscan1 0.5 system (RS Scan International, Belgium).	Kinetic variables: COP mean velocities: AP and ML directions (m/s)	Complexity index
Rathleff et al., 2013 [22]	Electronic uniaxial goniometer (BioVision, Werheim, Germany): placed around the tibia and femur.Two-foot switches: 1 under the heel and 1 under the halluxElectromyography (BioVision, Werheim, Germany): bipolar surface electrodes (Ambu A/S, Neuroline, Ballerup, Denmark) were placed on the muscle bellies of VM and VL with an interelectrode distance of 2 cm.A portable handheld dynamometer (Power track II commander, Chiroform, Viborg, Denmark) was positioned perpendicularly to the anterior aspect of the tibia, 5 cm proximal to the medial malleolus.	Spatiotemporal parameters: Cadence (steps/m) Temporal parameters: Stance phase time (s) Joint kinematic: Knee (º) Electromyography activity: Vastus medialis Vastus lateralis (Magnitude by root mean square (mV))Kinetic variables: Maximal quadriceps torque (Nm)	Sample Entropy (SEn): values of sEMG from vastus medialis and vastus lateralis muscles at the start, middle, and end of the stair descent.
Barbosa et al., 2015 [28]	Speedometer (Swim speedometer, Swimsportec, Hildesheim, Germany), placed on the forehead wall of the swimming pool, about 0.2 m above the water surface. Its cable was attached to the swimmer’s hip.	Spatiotemporal parameters: Swimming velocity (m/s) Horizontal velocity of the hip (m/s) Speed fluctuation (dimensionless) Temporal parameters Stroke length (m) Stroke frequency (dimensionless)	**Temporal structure measures:** Approximate entropy
Bisi and Stagni, 2016 [24]	Two tri-axial wireless inertial sensors (OPALS, Apdm, USA): 1 placed on the lower back and 1 placed on the right leg.	Spatiotemporal parameters: Trunk acceleration (m/s^2^)—vertical (V), anteroposterior (AP), and mediolateral (ML) components Right leg acceleration (m/s^2^)	Multiscale entropy (MSE): applied separately to the AP, vertical (V), and ML direction of the collected trunk acceleration (SEnV, SEnAP, and SEnML)
Vicinanza et al., 2018 [30]	Two 3D motion capture systems (CODA) sampling at 100 Hz (CODAmotion, Charnwood Dynamics Ltd., UK).Active markers were placed on the lateral aspect of each participant’s right side:mid forearm, greater trochanter, femoral condyle, lateral malleolus, fifth metatarsophalangeal, and the centre of the underside of the bar.	Joint kinematic: Shoulder, elbow, hip, knee, ankle, and foot (º)	Correlation dimension Determinism: Poincaré and Recurrent Quantification Analysis (RQA) Frequency analysis: Simpson entropy
Bisi and Stagni, 2018 [25]	Two tri-axial wireless inertial sensors (OPALS, Apdm, USA): 1 placed on the lower back and 1 placed on the right leg.	Spatiotemporal parameters: Trunk Acceleration (m/s^2^), for TW and NW: V, AP, and ML components	MSE: applied separately to the AP, V, and ML direction of the collected trunk acceleration (SEnV, SEnAP, and SEnML), in both NW and TW
Hamacher et al., 2018 [21]	Six inertial sensors (MTw2, Xsens Technologies B.V., Enschede, The Netherlands): 1 placed on the athletes’ back (T1), 1 placed on mid of a kayak ergometer paddle, 1 placed on the dorsa of each hand, and 1 placed on the mid of each upper arm.	Joint Kinematic: Hands, upper arm, and trunk (º) Spatiotemporal parameters: Hands, trunk, arms, and paddle angular velocity (m/s)	Largest Lyapunov exponent (using Rosenstein et al.’s algorithm)
Bartolomeu et al., 2018 [29]	Speedometer (swim speedometer, Swimsportec, Hildesheim, Germany), placed on a starting block in the headwall of the swimming pool. Its cable was attached to the swimmer’s hip.	Spatiotemporal parameters: Swimming velocity (m/s) Speed fluctuation (dimensionless)	SEn Fractal dimension
Bisi et al., 2019 [26]	Three tri-axial wireless inertial sensors (OPALS, Apdm, USA): 1 placed on the lower back (L5 level) and 1 placed on each shank (above lateral malleolus).	Temporal parameters: Stride time (s) Stance time (% stride time) Double support time (% stride time) Fundamental frequency (Hz)	Short Lyapunov exponent (sLe): sLeV, sLeML, and sLeAP MSE: calculated by assessing SEn on the 3 acceleration components (SEnV, SEnML, and SEnAP) RQA: recurrence rate, determinism, and averaged diagonal line length for each acceleration component (calculated on AP and ML directions) Poincaré Plots

DFA: Detrended fluctuation analysis; COP: centre-of-pressure; AP: anteroposterior; ML: mediolateral; SEn: sample entropy; sEMG: surface electromyography; MSE: multiscale entropy; sLe: short Lyapunov exponent; RQA: recurrent quantification analysis.

## 4. Discussion

This scoping review summarized the body of literature concerned with the biomechanical data of human movement, and the data were processed and analysed using nonlinear measures, such as innovative tools, to characterize different aspects of motor control performances, namely variability, stability, and the complexity of movement.

The gathered information allows a deeper understanding of how research has been conducted in real contexts among adolescents and which tasks, instruments, kinetic and kinematic variables, and nonlinear measures have been used in this field.

There is a lack of uniformity in the nomenclature used to describe the nonlinear measures in the included studies. This fact makes the interpretation and comparison of the data difficult. Considering that the study of the complexity of daily life tasks is a key point in human movement analyses, and it is crucial to adopt intervention strategies centred on nonlinear approaches, given that the human being’s movement is nonlinear, this lack of standardization of concepts may hinder the translation of these findings into human movement knowledge. This is a barrier to the implementation of a practice based on recent evidence, which makes us look at human movement as something that is highly complex, nonlinear, and endowed with variability.

### 4.1. Nonlinear Measures and Tasks

Given the complexity of human movement and since its variability translates into functionality, the analysis of a task using a combination of different nonlinear measures is important, as it will provide information about the different characteristics of movement variability [31]. Among the studies included in the review, the synthesis showed entropy as a measure that is mostly reported in kinetic and kinematic data processing (with an emphasis on MSE), followed by fractal analysis.

Entropy is a probabilistic complexity measure used in physiological signal analysis to quantify a time series’ irregularity [32]. While Costa et al. [12] identified the ApE as the most important entropy measure to assess kinetics and kinematics parameters in children and adolescents up to the age of 14, our review identified MSE as the most common measure of entropy reported in the included studies. In comparison with previous entropy measures, such as SEn or ApE (both identified alone in this review), MSE stands out due to the fact that it permits the assessment of complexity at shorter and longer time scales relative to the quantification of the overall complexity of a system [33,34]. It is known that ApE and SEn and their variants assess entropy only on a time scale, which seems to be insufficient for conveniently detailing physiological signals [10]. Thus, the choice of MSE in most of the studies included in our review seems to indicate a growing concern in the use of measures that can better reflect the complexity of movement, even in a more non-controlled environment, such as out-of-laboratory assessments [33].

Regardless of the applicability of the MSE, it is important to note that, in the studies included in this review, SEn and ApE were used when the data were collected in an aquatic environment. The ApE was used to quantify the regularity of fluctuations over the time series data [28], although the authors themselves stated that this measure has not been used previously to assess competitive swimming or any other competitive techniques. The SEn was applied to provide insight into the randomness of the intra-cyclic variations over the time series [29]. Preatoni et al. [35] suggested that these measures can be considered particularly appropriate for the study of sports movements, where variability is likely to have both a deterministic and a stochastic origin. Rathleff et al. [22], on the other hand, reported that SEn was used as an indicator of the complexity of the surface electromyography (sEMG) time series during stair walking movements. Based on the above-mentioned studies, it seems that the term complexity acquired synonyms, such as “randomness” or “regularity”, in accordance with the research question of the studies. This single- scale entropy analysis can be used to quantify regularity/predictability/probability/randomness; however, they do not capture the structural richness and wide-range component characterization of a complex system operating across multiple spatial and temporal scales [36].

Regarding the use of entropy measures, fractal analysis is another main nonlinear measure to highlight. While the included studies that used fractal analysis focus on different tasks, such as gait, swimming, and long swing, it seems to share common ground with the comparison of the same fractal characteristics. Since it is known that fractal analysis aims to quantify self-similarity and fractal or multifractal-like behaviours [37], this choice seems fully justified by the fact that it exhibits a statistical probability of self-similarity and, therefore, fractal-type behaviour. In two studies, fractal analyses, specifically fractal dimension and correlation dimension, were combined with entropy or RQA measurements, respectively. The fractal dimension was calculated by using Higuchi’s algorithm, and it is a suitable measure for time series data analyses, providing information about the intra-cyclical complexity and the irregularity of the variations in a given series [29]. Bartolomeu et al. [29] showed that swimming exhibits nonlinear properties and that the fractal dimension differs depending on the style of swimming and the level of specialization of the athlete. On the other hand, Vicinanza [30] applied the correlation dimension to calculate the fractal dimension of a time series in gymnastics [38]; moreover, while the participants looped the high bar, the findings showed that the dynamical degrees of freedom of the centre-of-mass in the skilled performance were reduced compared to those of novices, representing a more efficient and predictive technique rather than an exploratory one. Therefore, it seems that this measure can contribute to an improved understanding of the level of complexity of a cyclic movement that a subject develops relative to a specific skill in clinical practice.

Concerning the quantification of the local dynamic stability of complex nonlinear systems, the LLyE, which allows quantifying the rate of trajectory convergence or divergence in an n-dimensional state phase, was the measure applied in walking (NW and TW) [26] and performing paddling [21]. Both studies calculated the LyE using the algorithm of Rosenstein [39], which is the most frequently used algorithm in biomechanical studies [40]. Indeed, a review on gait demonstrated that 79% of the studies among young participants used Rosenstein’s method to calculate the LyE [41]. Raffalt et al. [42] described that its effectiveness is highly dependent on the applied times series normalization procedure, which did not happen in this study during the walking analysis [26]. Bisi, Tamburini, and Stagni [26], in order to complement the LyE and entropy measures, also applied the RQA to quantify the pattern regularities on NW and TW by using the calculation of the recurrence rate (its simplest measure) [43], determinism (which reflects the predictability/regularity of a time series) [44], and averaged diagonal line length. Assuming that the temporal gait parameters are nonlinear, nonstationary, and noisy by nature, and the RQA does not rely on assumptions, such as nonlinearity, nonstationarity, and noiselessness, and works well on short-length gait time series [45], this nonlinear measure helps us understand the periodicity and randomness of the gait.

### 4.2. Assessment Instruments and Kinematic and Kinetic Variables

Inertial measurement units (IMUs) were the preferred instruments [21,24,25,26] used to evaluate the kinematic data during walking or paddling. Moreover, foot switches were used during walking [23] or stair descent walking [22]. The motion capture system was used only when the data collection was carried out in a training centre [30]. Currently, it is known that wearable systems allow physiotherapists and other professionals working with movement to assess human movement in a more robust, rigorous, valid, and reliable manner in a real context [46]. The number of IMUs or their placement was not consistent between the studies; however, in the gait analysis, the right leg and upper/lower back were always analysed. It should be noted that none of these articles presented an explanation for the choice of the right leg for the placement of the IMU. However, it is known that as gait is a symmetrical activity, the use of one sensor only allows us to improve mobility and reduces power consumption [47].

A higher variety of kinematic outcomes was observed, and as expected, the most pointed outcomes were related to spatiotemporal parameters. Some authors propose combining these variables with joint kinematics or temporal parameters. Indeed, the sensor-based movement analysis generates a large volume of kinematic data for the sagittal, transverse and frontal planes, and joints simultaneously [48].

Regarding kinetic data, the studies included in this review analysed the COP mean velocity and maximal quadriceps torque using a force plate [27] and portable handheld dynamometers [22], respectively. The force plate is a gold standard in the kinetic analysis of human movement in several functional tasks, and it is a reference in the comparison of measurements obtained with other instruments and, therefore, is frequently used in scientific research [49,50,51]. However, given the characteristics of the force plates, its use was only possible because the data collection took place at the adolescents’ school. These data reinforce the difficulty of collecting kinetic data in a real context, and in fact, only two studies focused on the kinetics of human movement [22,27].

Some limitations should be highlighted. First, the search was limited to four databases. Hence, we cannot exclude the possibility of having missed some of the relevant literature. Second, we deliberately defined a board search strategy to minimize the risk of not identifying key papers. However, it may have restricted the initially identified studies. Moreover, there was significant heterogeneity between studies in the nomenclature used to describe nonlinear measures; therefore, our results must be interpreted cautiously.

## 5. Conclusions

This review demonstrated that, in adolescents assessed in a real context, entropy measures are the preferred ones when studying the complexity of human movement, especially when examining multiscale entropy. Over the years, authors have shown care in combining different measures, namely entropy measures and fractal analysis.

The non-laboratory contexts identified were schools and training centres (either on the ground or in aquatic environments). The kinematics of human movement has been the subject of more studies compared to kinetics, with a focus on walking.

### Future Directions

Despite the interesting studies included in this review, there are significant gaps in knowledge that remain in the literature on adolescent movement analyses that benefit from additional research. Gait is the most-studied task; however, there is a wide range of tasks, complex in itself, that was not studied in a real context. Exploratory studies assessing tasks, such as reaching, sit-to-stand, and stand-to-sit, which could contribute to an improved understanding and monitoring of motor development in adolescence due to their representativeness in daily life, are clearly needed.

Furthermore, although the world of nonlinear measurements is continuously growing, the definition of the objectives of the studies centred on the standardization of concepts can be presented as a suggestion for future investigations. Well-designed studies with standardised concepts, measures, and assessment protocols are needed for a better translation of knowledge into clinical practice. We also suggest research focusing on the kinetics of human movement.

## Figures and Tables

**Table 1:** Eligibility criteria according to PCC.

Criteria	
**Population**	Healthy teenagers between 10 and 19 years old [16]. Adolescents were also considered eligible regardless of whether they were in an experimental or control group or when the studies were experimental or quasi-experimental.
**Concept**	Nonlinear measurements in kinetic and/or kinematic data processing of human movement.
**Context**	Assess human movement out of the laboratory, i.e., non-laboratory settings; free living, daily living, or real-life environments.

## Data Availability

Not applicable.

## Supplementary Materials

The following supporting information can be downloaded at: https://www.mdpi.com/article/10.3390/s23010304/s1, Table S1: Initial search for different databases.

## References

[1] Bernshtein N.A. (1967). The Co-Ordination and Regulation of Movements.

[2] Hadders-Algra M. (2018). Early human motor development: From variation to the ability to vary and adapt. Neurosci. Biobehav. Rev..

[3] Stergiou N., Decker L.M. (2011). Human movement variability, nonlinear dynamics, and pathology: Is there a connection?. Hum. Mov. Sci..

[4] Stergiou N., Harbourne R.T., Cavanaugh J., Cavanaugh J. (2006). Optimal movement variability: A new theoretical perspective for neurologic physical therapy. J. Neurol. Phys. Ther..

[5] Latash M.L., Danion F., Scholz J.F., Zatsiorsky V.M., Schöner G. (2003). Approaches to analysis of handwriting as a task of coordinating a redundant motor system. Hum. Mov. Sci..

[6] Latash M.L., Scholz J.P., Fau-Schöner G., Schöner G. (2002). Motor control strategies revealed in the structure of motor variability. Exerc. Sport Sci. Rev..

[7] Korn H., Faure P. (2003). Is there chaos in the brain? II. Experimental evidence and related models. C. R. Biol..

[8] Cavanaugh J., Stergiou N., Kelty-Stephen D. (2017). Multifractality, Interactivity, and the Adaptive Capacity of the Human Movement System: A Perspective for Advancing the Conceptual Basis of Neurologic Physical Therapy. J. Neurol. Phys. Ther..

[9] Harbourne R.T., Stergiou N. (2009). Movement variability and the use of nonlinear tools: Principles to guide physical therapist practice. Phys. Ther..

[10] van Emmerik R.E.A., Ducharme S.W., Amado A.C., Hamill J. (2016). Comparing dynamical systems concepts and techniques for biomechanical analysis. J. Sport Health Sci..

[11] Bruijn S.M., Meijer O.G., Beek P.J., van Dieën J.H. (2013). Assessing the stability of human locomotion: A review of current measures. J. R. Soc. Interface.

[12] da Costa C.S.N., Batistão M.V., Rocha N.A.C.F. (2013). Quality and structure of variability in children during motor development: A systematic review. Res. Dev. Disabil..

[13] Lanovaz J.L., Oates A.R., Treen T.T., Unger J., Musselman K.E. (2017). Validation of a commercial inertial sensor system for spatiotemporal gait measurements in children. Gait Posture.

[14] Newell K.M., Vaillancourt D.E., Sosnoff J.J., Birren J.E., Schaie K.W., Abeles R.P., Gatz M., Salthouse T.A. (2006). Eight-Aging, Complexity, and Motor Performance. Handbook of the Psychology of Aging.

[15] Newell K.M., Vaillancourt D.E. (2001). Dimensional change in motor learning. Hum. Mov. Sci..

[16] Bundy D.A.P., de Silva N., Horton S., Patton G.C., Schultz L., Jamison D.T., Abubakara A., Ahuja A., Alderman H., Allen N. (2018). Investment in child and adolescent health and development: Key messages from Disease Control Priorities, 3rd Edition. Lancet.

[17] Tricco A.C., Lillie E., Zarin W., O’Brien K.K., Colquhoun H., Levac D., Moher D., Peters M.D.J., Horsley T., Weeks L. (2018). PRISMA Extension for Scoping Reviews (PRISMA-ScR): Checklist and Explanation. Ann. Intern. Med..

[18] Peters M., Marnie C., Tricco A.C., Pollock D., Munn Z., Alexander L., McInerney P., Godfrey C.M., Khalil H. (2020). Updated methodological guidance for the conduct of scoping reviews. JBI Evid. Synth..

[19] Aromataris E., Munn Z. (2020). Furthering the science of evidence synthesis with a mix of methods. JBI Manual Evid. Synth..

[20] Page M.J., McKenzie J.E., Bossuyt P.M., Boutron I., Hoffmann T.C., Mulrow C.D., Shamseer L., Tetzlaff J.M., Akl E.A., Brennan S.E. (2021). The PRISMA 2020 statement: An updated guideline for reporting systematic reviews. BMJ.

[21] Hamacher D., Krebs T., Meyer G., Zech A. (2018). Does local dynamic stability of kayak paddling technique affect the sports performance? A pilot study. Eur. J. Sport Sci..

[22] Rathleff M.S., Samani A., Olesen J.L., Roos E.M., Rasmussen S., Christensen B.H., Madeleine P. (2013). Neuromuscular activity and knee kinematics in adolescents with patellofemoral pain. Med. Sci. Sports Exerc..

[23] Hausdorff J.M., Zemany L., Fau-Peng C., Peng C., Fau-Goldberger A.L., Goldberger A.L. (1999). Maturation of gait dynamics: Stride-to-stride variability and its temporal organization in children. J. Appl. Physiol. (1985).

[24] Bisi M.C., Stagni R. (2016). Complexity of human gait pattern at different ages assessed using multiscale entropy: From development to decline. Gait Posture.

[25] Bisi M.C., Stagni R. (2018). Changes of human movement complexity during maturation: Quantitative assessment using multiscale entropy. Comput. Methods Biomech. Biomed. Eng..

[26] Bisi M.C., Tamburini P., Stagni R. (2019). A ‘Fingerprint’ of locomotor maturation: Motor development descriptors, reference development bands and data-set. Gait Posture.

[27] Pau M., Kim S., Nussbaum M.A. (2012). Does load carriage differentially alter postural sway in overweight vs. normal-weight schoolchildren?. Gait Posture.

[28] Barbosa T.M., Morais J.E., Marques M.C., Silva A.J., Marinho D.A., Kee Y.H. (2015). Hydrodynamic profile of young swimmers: Changes over a competitive season. Scand. J. Med. Sci. Sports.

[29] Bartolomeu R.F., Costa M.J., Barbosa T.M. (2018). Contribution of limbs’ actions to the four competitive swimming strokes: A nonlinear approach. J. Sports Sci..

[30] Vicinanza D., Newell K.M., Irwin G., Smith L., Williams G.K.R. (2018). Limit cycle dynamics of the gymnastics longswing. Hum. Mov. Sci..

[31] Caballero C., Barbado D., Moreno F.J. (2014). Non-linear tools and methodological concerns measuring human movement variability: An overview. Eur. J. Hum. Mov..

[32] Ribeiro M., Henriques T., Castro L., Souto A., Antunes L., Costa-Santos C., Teixeira A. (2021). The Entropy Universe. Entropy.

[33] Busa M.A., van Emmerik R.E.A. (2016). Multiscale entropy: A tool for understanding the complexity of postural control. J. Sport Health Sci..

[34] Gruber A.H., Busa M.A., Gorton Iii G.E., Van Emmerik R.E.A., Masso P.D., Hamill J. (2011). Time-to-contact and multiscale entropy identify differences in postural control in adolescent idiopathic scoliosis. Gait Posture.

[35] Preatoni E., Ferrario M., Donà G., Hamill J., Rodano R. (2010). Motor variability in sports: A non-linear analysis of race walking. J. Sports Sci..

[36] Yentes J.A.-O., Raffalt P.C. (2021). Entropy Analysis in Gait Research: Methodological Considerations and Recommendations. Ann. Biomed. Eng..

[37] Ribeiro M., Monteiro-Santos J., Castro L., Antunes L., Costa-Santos C., Teixeira A., Henriques T.S. (2021). Non-linear Methods Predominant in Fetal Heart Rate Analysis: A Systematic Review. Front. Med..

[38] Barabási A.L., Stanley H.E. (1995). Fractal Concepts in Surface Growth.

[39] Rosenstein M.T., Collins J.J., De Luca C.J. (1993). A practical method for calculating largest Lyapunov exponents from small data sets. Phys. D Nonlinear Phenom..

[40] Mehdizadeh S., Sanjari M.A. (2017). Effect of noise and filtering on largest Lyapunov exponent of time series associated with human walking. J. Biomech..

[41] Mehdizadeh S. (2018). The largest Lyapunov exponent of gait in young and elderly individuals: A systematic review. Gait Posture.

[42] Raffalt P.C., Kent J.A., Wurdeman S.R., Stergiou N. (2019). Selection Procedures for the Largest Lyapunov Exponent in Gait Biomechanics. Ann. Biomed. Eng..

[43] Marwan N., Carmen Romano M., Thiel M., Kurths J. (2007). Recurrence plots for the analysis of complex systems. Phys. Rep..

[44] Chatain C., Ramdani S., Vallier J.-M., Gruet M. (2021). Recurrence quantification analysis of force signals to assess neuromuscular fatigue in men and women. Biomed. Signal Proc. Control.

[45] Prabhu P., Karunakar A.K., Anitha H., Pradhan N. (2020). Classification of gait signals into different neurodegenerative diseases using statistical analysis and recurrence quantification analysis. Pattern Recognit Lett..

[46] Drapeaux A., Carlson K. (2020). A Comparison of Inertial Motion Capture Systems: DorsaVi and Xsens. IJKSS.

[47] Ancillao A., Tedesco S., Barton J., O’Flynn B. (2018). Indirect Measurement of Ground Reaction Forces and Moments by Means of Wearable Inertial Sensors: A Systematic Review. Sensors.

[48] Benedetti M.G., Beghi E., De Tanti A., Cappozzo A., Basaglia N., Cutti A.G., Cereatti A., Stagni R., Verdini F., Manca M. (2017). SIAMOC position paper on gait analysis in clinical practice: General requirements, methods and appropriateness. Results of an Italian consensus conference. Gait Posture.

[49] García-Ramos A., Štirn I., Padial P., Argüelles-Cienfuegos J., De la Fuente B., Strojnik V., Feriche B. (2015). Predicting vertical jump height from bar velocity. J. Sports Sci. Med..

[50] Harro C.C., Garascia C. (2019). Reliability and Validity of Computerized Force Platform Measures of Balance Function in Healthy Older Adults. J. Geriatr. Phys. Ther..

[51] Pontillo M., Hines S.M., Sennett B.J. (2021). Prediction of ACL Injuries from Vertical Jump Kinetics in Division 1 Collegiate Athletes. Int. J. Sports Phys. Ther..

