# Supplementary material for: Methodological Considerations in the Kinematic and Kinetic Analysis of Human Movement among Healthy Adolescents: A Scoping Review of Nonlinear Measures in Data Processing
Source: Sensors (Basel). 2022 Dec 28;23(1):304. doi: 10.3390/s23010304 (PMC9823368; doi:10.3390/s23010304)
Supplement: Supplementary file 1 [file sensors-23-00304-s001.zip › sensors-2050265-supplementary.pdf]

Methodological considerations in kinematic and kinetic analysis of human movement among healthy adolescents: Protocol for a scoping review of nonlinear measures in data processing

Prepared for Registration to Open Science Framework

Submitted 16/03/2022

Departamento de Tecnologias de Diagnóstico e Terapêutica
CESPU: Escola Superior de Saúde do Vale do Ave
Vila Nova de Famalicão, Portugal

Sandra Silva
sandra.silva@ipsn.cespu.pt

REVIEW TITLE

Methodological considerations in kinematic and kinetic analysis of human movement among healthy adolescents: Protocol for a scoping review of nonlinear measures in data processing

TYPE OF REVIEW

Scoping Review.

STAGE OF REVIEW AT TIME OF THIS SUBMISSION

Ongoing: Piloting of the study selection process.

ORGANISATIONAL AFFILIATION OF THE REVIEW

CESPU: Escola Superior de Saúde do Vale do Ave

University of Aveiro

REVIEW TEAM MEMBERS AND AFFILIATION

Sandra Silva
Escola Superior de Saúde do Vale do Ave (ESSVA), Cooperativa de Ensino Superior Politécnico e Universitário (CESPU), Vila Nova de Famalicão, Portugal; School of Health Sciences (ESSUA) and Department of Medical Sciences (DCM), University of Aveiro, Aveiro, Portugal. Electronic address: sandra.silva@ipsn.cespu.pt

Vânia Figueira
Escola Superior de Saúde do Vale do Ave (ESSVA), Cooperativa de Ensino Superior Politécnico e Universitário (CESPU), Vila Nova de Famalicão, Portugal; Research Centre in Physical Activity, Health and Leisure, Faculty of Sport, University of Porto, Porto, Portugal.

Fernando Ribeiro
Institute of Biomedicine - iBiMED, School of Health Sciences, University of Aveiro, Aveiro, Portugal.

Francisco Pinho
Escola Superior de Saúde do Vale do Ave (ESSVA), Cooperativa de Ensino Superior Politécnico e Universitário (CESPU), Vila Nova de Famalicão, Portugal.

REVIEW METHODS

This scoping review will be conducted in accordance with the Preferred Reporting Items for Systematic Reviewers and Meta-Analysis extension for Scoping Reviews (PRISMA-ScR) framework (Tricco et al, 2018).

Review questions

The main review question is "What are the nonlinear measures used in processing kinematic and kinetic data in the assessment of human movement among healthy adolescents?"

The review sub-questions are:

1. What instruments were used to collect kinematic and kinetic data in the identified studies?
2. What kinematic and kinetic variables were considered in the identified studies?
3. What tasks were covered in the identified studies?

Eligibility Criteria

Eligibility criteria were established *a priori* using the acronym PCC (Population, Concept, Context), in accordance with Joanna Briggs Institute methodology (Peters et al., 2020).

Population: Healthy teenagers between 10 and 19 years old.

Concept: Report nonlinear measurements in kinetic and/or kinematic data processing of human movement.

Context: Assess human movement out of the laboratory, i.e., non-laboratory settings; free-living, daily-living or real-life environments.

Studies are also eligible if they meet the following criteria:

- Experimental and epidemiological study designs.

- Studies published in Portuguese, French, English and Spanish.

Studies will be excluded if they have any of the following characteristics:

- Systematic, narrative or scoping reviews will not be included to avoid duplication of data.

- Qualitative method designs.

Information Source

The relevant studies will be identified by searching the databases PubMed, Science Direct and Web of Science since their inception until March 2022. The Google Scholar will be contemplated as unpublished and gray literature. The reference lists of original research articles and reviews on the topic will be manually checked to identify other eligible studies. The search strategy for different databases is presented in Table S1, according to their specificities. Two reviewers, independently, will carry out the search.

Table S1: Initial search for different databases

Database	Entry Terms
PubMed	((nonlinear measures) OR (nonlinear dynamics) OR (entropy) OR (motor variability)) AND ((adolescents) OR (children)) AND ((kinematic) OR (kinetic)).
Science Direct	("nonlinear measures" OR "nonlinear dynamics" OR "entropy" OR "motor variability") AND (adolescents OR children) AND (Kinematic OR kinetic)
Web of Science	("nonlinear measures" OR "entropy" OR "nonlinear dynamics" OR "movement variability" OR "movement complexity") AND (adolescent OR children) AND (Kinematic OR kinetic)
Google Scholar	((("nonlinear measures") OR ("nonlinear dynamics") OR ("entropy") OR ("motor variability")) AND (("healthy adolescents") OR ("healthy children")) AND ((kinematic) OR (kinetic))) AND ("real life" OR "real time" OR "out of laboratory")

Selection of Evidence Sources

The selection of evidence sources will consider the PCC acronym, purpose and research questions. The data will be extracted by two independent reviewers and any disagreements between them will be resolved through discussion or with a third reviewer (if a consensus is not reached).

A pilot testing will be carried out, where all reviewers will analyze the same 25 publications (the first 25 titles/abstracts of PubMed database). Based on eligibility criteria defined a priori, an analysis of titles/abstracts will be carried out independently by the 2 reviewers. After discussion of the results of this calibration exercise and consensus has been reached, changes will be made to

the eligibility criteria if necessary. The researchers will start the screening process only when there is a consensus of at least 75%.

After the search, all identified records will be imported to Endnote software (Clarivate) and duplicates will be removed. The titles and abstracts will be screened by two reviewers, after which they will categorize studies as "include" or "exclude". This stage will allow identifying articles for full-text screening. The reasons why the studies were excluded will be detailed in the final scoping review.

The results of the search will be presented in a PRISMA-ScR flowchart.

Data Extraction

Data will be extracted for the authors, year of publication, study design, participant characteristics (n, age, % female, BMI), the tasks under study, assessment instruments to capture the human body motion, outcome kinetic and kinematic measures, sampling method and the study setting. If necessary, the corresponding authors of published articles will be contacted by e-mail to request missing or additional data. Two authors will independently extract the abovementioned data by using a draft charting table adapted from the original JBI template. The data extraction will be performed independently by the 2 reviewers, while disagreements will be resolved with a third author.

Critical appraisal of individual sources of evidence

We will not conduct quality appraisal, since this is a scoping review, which is in accordance with Preferred Reporting Items for Systematic reviews and Meta-Analyses extension for Scoping Reviews and Joanna Briggs Institute methodology.

Data Presentation

The full scoping review will be reported in accordance with the PRISMA-ScR checklist.

A narrative report will be produced to summarize the extracted data around the following outcomes: nonlinear measures, tasks, kinematic and kinetic variables and instruments. These

results will be described in relation to the research question and in the context of the overall study purpose. Gap identification will detect areas, such as tasks and contexts that lack data on the nonlinear measures to report kinematic and kinetic data, and if there is limited data among healthy adolescents. To summarize the evidence on specific topics, all the results will be presented in 2 tables, by two main conceptual categories, the kinetic and kinematic data. A synthesis of the main findings will accompany the tabulated data.

KEYWORDS

Nonlinear Variables; Adolescents, Out-Of-Laboratory, Variability, Biomechanics

AUTHOR´S CONTRIBUTIONS

Authors SS and FP conceived the idea of the scoping review and developed the research questions. FP provided conceptual expertise of the nonlinear measures. SS, FR and FP contributed meaningfully to drafting and editing the manuscript and developing the study methods. SS and VF contributed to the search strategies and created the search strategy table.

FUNDING SOURCES

None.

CONFLICT OF INTEREST

None Declared.

COUNTRY

Portugal
